# Estimating the optimal rate of adjuvant chemotherapy utilization for stage III colon cancer

**DOI:** 10.1002/cam4.2456

**Published:** 2019-08-12

**Authors:** Safiya Karim, Christopher M. Booth, Kelly Brennan, Yingwei Peng, D. Robert Siemens, Monika K. Krzyzanowska, William J. Mackillop

**Affiliations:** ^1^ Department of Oncology, Tom Baker Cancer Centre University of Calgary Calgary AB Canada; ^2^ Departments of Oncology Queen's University Kingston ON Canada; ^3^ Departments of Public Health Sciences Queen's University Kingston ON Canada; ^4^ Division of Cancer Care and Epidemiology Queen's University Cancer Research Institute Kingston ON Canada; ^5^ Departments of Urology Queen's University Kingston ON Canada; ^6^ Division of Medical Oncology and Hematology Princess Margaret Cancer Centre Toronto OV Canada; ^7^ Department of Medicine University of Toronto Toronto ON Canada

## Abstract

**Background:**

Identifying optimal chemotherapy utilization rates can drive improvements in quality of care. We report a benchmarking approach to estimate the optimal rate of adjuvant chemotherapy (ACT) for stage III colon cancer.

**Methods:**

The Ontario Cancer Registry and linked treated records were used to identify ACT utilization. Monte Carlo simulation was used to estimate the proportion of ACT rate variation that could be due to chance alone. The criterion‐based benchmarking approach was used to explore whether socioeconomic or system‐level factors were associated with ACT. We also used the “pared‐mean” approach to identify a benchmark population of hospitals with the highest ACT rates.

**Results:**

The study population included 2801 patients; ACT was delivered to 66% (1861/2801). Monte Carlo simulation suggested that the observed component of variation (15.6%) in ACT rates was within the 95% CI (11.5%‐17.3%) of what could be expected due to chance alone; the nonrandom component of ACT rate variation across hospitals was only 1.5%. There was no difference in hospital ACT rate by teaching status (*P* = .107), cancer center status (*P* = .362), or having medical oncology on site (*P* = .840). Unadjusted ACT rates varied across hospitals (range 44%‐91%, *P* = .017). The unadjusted benchmark ACT rate was 81% (95%CI 76%‐86%); utilization rate in non‐benchmark hospitals was 65% (95%CI 63%‐66%). However, after adjusting for case mix, the difference in ACT utilization between benchmark and non‐benchmark populations was significantly smaller.

**Conclusions:**

We did not find any system‐level factors associated with the utilization of ACT. Our results suggest that the observed variation in hospital ACT rate is not significantly different from variation due to chance alone. Using the “pared‐mean” approach may significantly overestimate optimal treatment rates if case mix is not considered.

## Graphical abstract

Identifying optimal chemotherapy utilization rates can drive improvements in quality of care. This benchmarking study of adjuvant chemotherapy (ACT) rates for stage III colon cancer showed that there were no system‐level factors associated with the utilization of ACT and that “pared‐mean” approach may significantly overestimate optimal treatment rates if case mix is not considered.

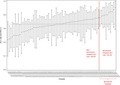

## 1 INTRODUCTION

Adjuvant chemotherapy (ACT) is the standard of care for stage III colon cancer and associated with an absolute improvement in survival of 15%‐20%.^1^, ^2^, ^3^ Despite robust evidence, it is well known that not all patients will receive guideline‐concordant care. Only 66% of patients in Ontario received adjuvant chemotherapy^4^; comparable rates have been reported elsewhere.^5^, ^6^, ^7^ Interpretation of this data is limited by the fact that the optimal utilization rate is not known. An evidence‐based model from 2009 estimated that 89% of patients with stage III colon cancer cases should receive ACT,^8^ but this likely overestimates the appropriate rate because the model did not consider the impact of comorbidity and patient preferences. Without knowing the optimal ACT utilization rate, it is not possible to identify shortfalls in utilization and therefore not possible to close the gap between evidence and practice.^10^

Two other methods have been widely used to estimate optimal treatment rates. Criterion‐based benchmarking (CBB) is an empirical method for estimating the appropriate rate of treatment based on direct observation of the rates actually achieved in settings where access to treatment is optimal.^11^ This approach has been used to estimate the proportion of patients who need radiotherapy.^12^, ^13^, ^14^ The University of Alabama at Birmingham's Achievable Benchmarks of Care (ABC^TM^s) is another empirical method that has been used for identifying benchmark performance for a variety of process‐of‐care indicators. This is achieved by calculating a “pared‐mean,” defined as the mean of the best care achieved for at least 10% of the population.^16^ This methodology is widely used in quality improvement projects.

The objective of our study was to describe variations in using ACT for stage III colon cancer in Ontario and to apply the CBB and ABC methodologies to estimate the appropriate rate.

## 2 METHODS

### 2.1 Study population

The study population included patients who underwent resection of stage III colon cancer in the Canadian province of Ontario during 2000‐2008. Ontario has a population of 13.5 million people and a single‐payer universal health care system. We used the Ontario Cancer Registry (OCR) to identify all incident cases of colorectal cancer diagnosed in Ontario. The OCR does not capture stage of disease for all patients; therefore, we obtained surgical pathology reports for a random sample of 25% of cases. Reports were not available for patients with surgery in 2005; therefore, the study cohort is restricted to patients who had surgery during 2000‐2004 and 2006‐2008 and we obtained a surgical pathology report. The study was approved by the Research Ethics Board at Queen's University, Kingston, Canada.

### 2.2 Data sources and linkages

The Ontario Cancer Registry (OCR) is a population‐based cancer registry that captures ~98% of all incident cases of cancer in the province.^17^, ^18^ Records of hospitalization from the Canadian Institute for Health Information (CIHI) provided information about surgical procedures; these records are known to have a very high level of completeness for colorectal cancer surgery.^19^ Physician billing records from the Ontario Health Insurance Plan and electronic records of treatment were used to identify chemotherapy utilization. Original reports of surgical pathology were obtained from the OCR and reviewed by a team of trained data abstractors.

### 2.3 Definition of variables

Teaching hospital was by affiliation with a medical school and routinely having residents on service. Hospitals that delivered chemotherapy were classified as having medical oncology on site. Ontario's regional cancer centres (RCC) are comprehensive cancer centers with on‐site radiation facilities; during the study period there were 13 RCCs.

Indicators of the socioeconomic status (SES) of the community in which patients resided were linked as described previously.^20^ Rurality was defined as living in a municipality with fewer than 10 000 people. Comorbidity was classified using the modified Charlson Index.^21^

### 2.4 Outcome measures

ACT rate was defined as the percentage of patients who started chemotherapy within 16 weeks of surgery. The degree of variation in using ACT in the province was described by the coefficient of variation (CV) of the hospital‐specific ACT rates. The CV is the ratio of the standard deviation to the mean of the ACT rates. When actual rates are lower than the benchmarks of the appropriate rate, the unmet need for ACT is measured in terms of the “shortfall,” where: %Shortfall = (benchmark rate‐actual rate)/benchmark rate × 100%.

### 2.5 Quantifying the random component of variation in ACT rates

Monte Carlo simulation was used to determine the degree of interhospital variation in ACT rates that would be expected due to chance alone, as described previously.^22^ The simulation model assumed that the probability of using ACT was the same at every hospital, and equal to the observed probability of using ACT in the overall study population. The model used the actual number of hospitals in Ontario and the actual number of patients seen at each hospital. We did 1000 iterations of the model. The CV of hospital‐specific ACT rates was calculated for each set of simulated data. The mean and 95% CI of the 1000 simulated CVs were used to quantify the degree of variation in hospital‐specific ACT rates that would be expected due to chance alone in this study population. The magnitude of the nonrandom component of variation in ACT rates was estimated by subtracting the expected CV from the observed CV.

### 2.6 The CBB method

The CBB process used had four steps: (a) logistic regression to identify social and health system‐related factors that impede access to ACT; (b) identification of a benchmark subpopulation with unimpeded access to ACT; (c) measurement of the ACT rate in the benchmark population; (d), direct standardization of the benchmark rate to the case mix of the general cancer population.^9^

### 2.7 The ABC method

The ABC method is operationalized using the “pared‐mean” approach. To create benchmark levels, hospitals were ranked on descending order of rates of ACT. We then removed those hospitals with <10 study cases over the study period. Beginning with the best performing hospital, the eligible patients in each hospital were then summed sequentially until the combined population size of this subset of hospitals was 10% of the entire study population. The benchmark rate was then calculated as the proportion of eligible patients in these top‐performing hospitals who received ACT.^16^ To account for differences in case mix across hospitals, we adjusted the observed hospital‐specific ACT rates for patient‐ and disease‐related factors using a parametric bootstrapping approach (Supplemental Appendix). All statistical analyses were carried out using SAS version 9.4 (SAS Institute, Cary, NC).

## 3 RESULTS

### 3.1 Study population

The study population included 2801 patients with resected stage III colon cancer (Figure S1, Table 1). Our previous analysis showed that older patients with greater comorbidity were less likely to receive ACT; surgeon/hospital volumes were not associated with ACT utilization.^4^ There were total of 72 hospitals where patients had surgery. Fourteen were classified as teaching hospitals, 13 were comprehensive cancer centers, and 32 were hospitals with medical oncology services on site. Twenty‐three percent (638/2801) of patients had surgery at a teaching hospital; 23% (639/2801) had surgery at a comprehensive cancer center. Fifty percent (1408/2801) had surgery at a hospital with on‐site medical oncology services.

**Table 1:** Characteristics of patients with stage III colon cancer treated with surgical resection in Ontario during 2002‐2008 (n = 2801)

Characteristic	No. (%)
**Patient‐related**	
Age, years	
20‐49	207 (7)
50‐59	437 (16)
60‐69	727 (26)
70‐79	872 (31)
80+	558 (20)
Sex	
Male	1462 (52)
Female	1339 (48)
SES by quintile*	
1	595 (21)
2	648 (23)
3	590 (21)
4	509 (18)
5	453 (16)
Charlson comorbidity score	
0	2267 (81)
1	313 (11)
2+	221 (8)
**Disease‐related**	
Grade	
Well‐moderately differentiated	2141 (76)
Poorly differentiated	610 (22)
Unstated	50 (2)
Lymphovascular invasion	
Yes	1378 (49)
No	1209 (43)
NA	214 (8)
T Stage	
T ≤ 1	43 (2)
T2	180 (6)
T3	1854 (66)
T4	724 (26)
N stage	
N1	1663 (59)
N2	1138 (41)
Lymph nodes harvest	
Mean	16.9
Median	15
≥ 12	2051 (73)
< 12	740‐745 (27)
Unknown	<6 (0)
Adjuvant chemotherapy	
Yes	1861 (66)
No	940 (34)

Note

As per Institute of Clinical Evaluative Sciences policy, cells were suppressed to ensure that precise small cell values cannot be determined.

* Socioeconomic status, Quintile 1 represents the communities where the poorest 20% of the Ontario population resided. SES data were not available for six patients.

### 3.2 Interhospital variation in ACT rates

**Figure 1:**
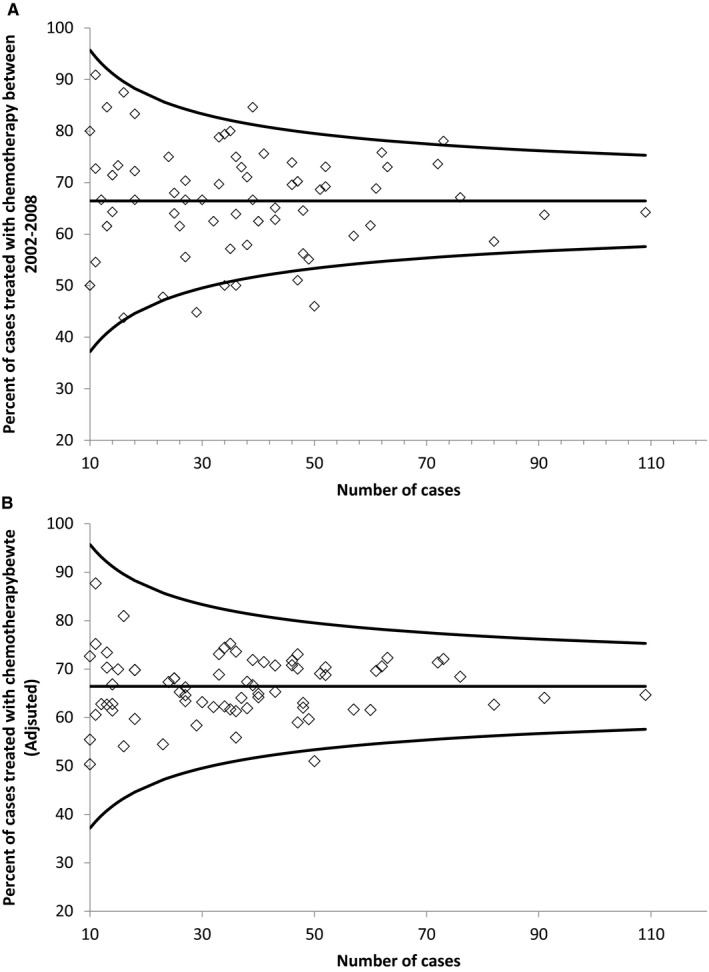
(A) Inter‐hospital variation in the use of adjuvant chemotherapy for stage III colon cancer in Ontario 2002‐2008. The provincial mean rate is the horizontal line, 95% confidence intervals are shown with the additional line. (B) Inter‐hospital variation in the adjusted adjuvant chemotherapy rate for stage III colon cancer in Ontario 2002‐2008. The provincial mean rate is the horizontal line, 95% confidence intervals are shown with the additional lines. Note: Hospitals with case volumes of <10 were excluded from this figure to ensure institutions cannot be identified. ◊ Each point represents one hospital

ACT was delivered to 66% (1861/2801) of all patients. Unadjusted hospital‐specific rates varied from 25% to 100% (IQR 62%‐73%) (Figure 1A). Figure 1B shows hospital‐specific rates after adjusting for patient‐ and disease‐specific factors that may influence the usage ACT. The observed coefficient of variation (CV) was 15.6%. However, at most individual hospitals, the observed ACT rate fell within the 95% CI of the province‐wide rate (Figure 1A,1). Moreover, Monte Carlo modeling showed that, if the underlying probability of ACT at each hospital was identical to the provincial rate, chance alone would lead to a similar degree of variation in hospital‐specific ACT rates, with the expected CV = 14.1% (95%CI 11.5%‐17.3%). Thus, the observed CV of 15.6% is only slightly higher than our best estimate of the CV expected due to chance alone, and lies well within the 95% CI of that estimate. The observed interhospital variations in ACT rates are therefore not necessarily indicative of systematic variations in practice.

### 3.3 CBB: System‐level factors associated with the usage of ACT

We hypothesized that decision‐making about the usage of ACT was most likely to be optimal in teaching hospitals, in comprehensive cancer centers, and in hospitals where medical oncologists were available on site. We tested those hypotheses in a multivariable logistic regression (Table 2); ACT rate was not associated with comprehensive cancer centers status or having medical oncology on site. Teaching hospitals were less likely to deliver ACT than nonteaching hospitals but this might be explained by unmeasured comorbidity. Thus, we did not find any health system‐related factors that explained the observed variations in using ACT. The absence of any specific explanation for the rate variations is consistent with our finding that most of the observed variation in ACT rates is random rather than systematic. In the absence of variation in ACT rates, the adjusted rates observed at comprehensive cancer centers are likely to approximate the optimal rates; therefore our criterion‐based benchmark rate would be 66%.

**Table 2:** The association between health system factors and delivery of adjuvant chemotherapy among patients with stage III colon cancer treated with surgery in Ontario 2002‐2008 (n = 2801)

Variable	Observed Adjuvant Chemotherapy Rate (Unadjusted) (%)	Unadjusted RR (95% CI)	Adjuvant chemotherapy rate (Adjusted*) (%)	Adjusted*RR (95%CI)
Model 1: Teaching Hospital				
No (n = 2163)	67	Ref	71	Ref
Yes (n = 638)	64	0.95 (0.89‐1.01)	64%	0.92 (0.87‐0.98)
Model 2: Comprehensive Cancer Center				
No (n = 2162)	67	Ref	70%	Ref
Yes (n = 639)	65	0.97 (0.91‐1.04)	66	0.96 (0.90‐1.01)
Model 3: On‐Site Medical Oncology				
No (n = 1393)	66%	Ref	69	Ref
Yes (n = 1408)	67	1.01 (0.95‐1.06)	70	1.01 (0.96‐1.05)

* Covariates in each adjusted model included: age, sex, socioeconomic status, Charlson comorbidity score, length of stay, Tstage, Nstage, lymphovascular invasion, and histological grade.

### 3.4 ABC: Achievable benchmarks of care

**Figure 2:**
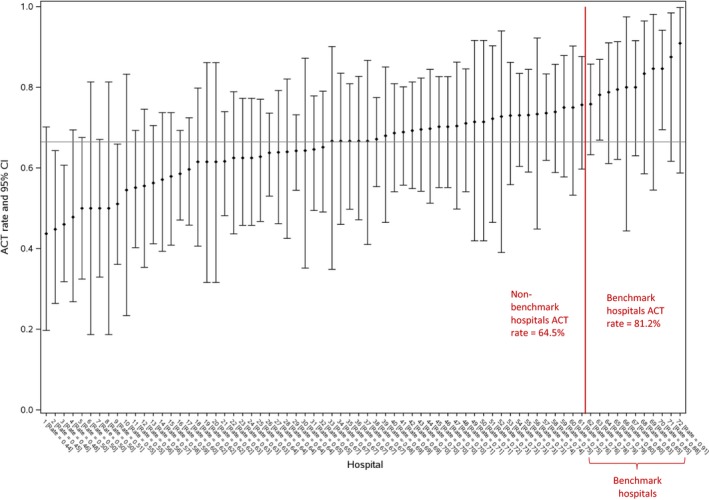
Hospital utilization rates of adjuvant chemotherapy for 2651 patients across 72 hospitals with stage III colon cancer treated in Ontario during 2002‐2008

After excluding 150 patients from hospitals with <10 cases, we applied the ABC method to the remaining 2651 patients. Characteristics of the 150 excluded patients were comparable to cases included in the ABC analysis (Table S1). The mean ACT rate across all hospitals was 66% (range 44%‐91%) (Figure 2). There were 282 patients in the 10 benchmark hospitals. Patient‐ and disease‐related characteristics of the benchmark and non‐benchmark populations were comparable (Table 3). None of the 10 benchmark hospitals were teaching hospitals or regional cancer centers; the benchmark hospitals were also smaller than non‐benchmark hospitals. The unadjusted ACT rate in the benchmark population was 81% (95% CI 76%‐86%) vs 65% (95% CI 63%‐66%) in the non‐benchmark population. If the rate of ACT in the benchmark population were accepted as the optimal rate, the % shortfall in the use ACT = [(81%‐66%)/81%] × 100% = 18.5%, indicating that on average we are missing the benchmark by almost one in five patients.

**Table 3:** Characteristics of patients with stage III colon cancer treated in Ontario during 2002‐2008 classified by hospital benchmark status

Characteristic	All Patients	Benchmark population	Non‐benchmark population	*P*
	N = 2651	N = 282	N = 2369	
	No. (%)	No. (%)	No. (%)	
**Patient‐related**				
Age (years)				.544
20‐49	195 (7%)	25 (9%)	170 (7%)	
50‐59	404 (15%)	39 (14%)	365 (15%)	
60‐69	690 (26%)	73 (26%)	617 (26%)	
70‐79	834 (31%)	96 (34%)	738 (31%)	
80+	528 (20%)	49 (17%)	479 (20%)	
Sex				.574
Female	1264 (48%)	130 (46%)	1134 (48%)	
Male	1387 (52%)	152 (54%)	1235 (52%)	
SES by quintile*				.157
1	561 (21%)	44 (16%)	517 (22%)	
2	600 (23%)	73 (26%)	527 (22%)	
3	557 (21%)	59 (21%)	498 (21%)	
4	485 (18%)	59 (21%)	426 (18%)	
5	442 (17%)	47 (17%)	395 (17%)	
Unknown	6 (0%)	0 (0%)	6 (0%)	
Charlson comorbidity score				.835
0	2150 (81%)	232 (82%)	1918 (81%)	
1	291 (11%)	30 (11%)	261 (11%)	
2+	210 (8%)	20 (7%)	190 (8%)	
Length of hospital stay (days)				
Median	8	8	8	.537
**Disease‐related**				
Grade				.085
Well‐moderately differentiated	2028 (76%)	201 (71%)	1827 (77%)	
Poor differentiated	575‐580 (22%)	75‐80 (27%)	500‐505 (21%)	
Unstated	43‐48 (2%)	≤5 (2%)	35‐40 (2%)	
Lymphovascular invasion				.341
No	1131 (43%)	127 (45%)	1004 (42%)	
Yes	1315 (50%)	139 (49%)	1176 (50%)	
NA	205 (8%)	16 (6%)	189 (8%)	
T stage				.024
pT ≤ 1	40 (2%)	7 (2%)	33 (1%)	
pT2	170 (6%)	22 (8%)	148 (6%)	
pT3	1764 (67%)	200 (71%)	1564 (66%)	
pT4	677 (26%)	53 (19%)	624 (26%)	
N stage				.293
N1	1568 (59%)	175 (62%)	1393 (59%)	
N2	1083 (41%)	107 (38%)	976 (41%)	
Lymph node harvest				.067
≥12	1960 (74%)	201 (71%)	1759 (74%)	
<12	685‐690 (26%)	75‐80 (28%)	605‐610 (26%)	
Unknown	≤5 (0%)	≤5 (1%)	≤5 (0%)	
**Hospital‐related**				
Teaching Hospital				<.001
No (n = 2013)	2013 (76%)	282 (100%)	1731 (73%)	
Yes (n = 638)	638 (24%)	0 (0%)	638 (27%)	
Regional Cancer Center				<.001
No (n = 2017)	2017 (76%)	282 (100%)	1735 (73%)	
Yes (n = 634)	634 (24%)	0 (0%)	634 (27%)	
Medical Oncologist on Site				.138
No (n = 1248)	1248 (47%)	121 (43%)	1127 (48%)	
Yes (n = 1403)	1403 (53%)	161 (57%)	1242 (52%)	

Note

As per Institute of Clinical Evaluative Sciences policy, cells were suppressed to ensure that precise small cell values cannot be determined.

* Socioeconomic status, Quintile 1 represents the communities where the poorest 20% of the Ontario population resided.

### 3.5 Adjusting for patient‐related variables that affect eligibility for ACT

The original ABC approach does not take into account the case mix of patients when determining a benchmark population. In order to improve upon this methodology, we adjusted the observed hospital‐specific ACT rates for patient‐ and disease‐related factors using a parametric bootstrapping approach (Supplemental Appendix). We reapplied the ABC methodology to the distribution of adjusted rates to identify an adjusted ABC benchmark population. The ACT rate in this adjusted benchmark population was 74% (95% CI 70%‐79%) vs 65% (95% CI 63%‐67%) in the non‐benchmark population. If the rate of ACT in the adjusted benchmark population were accepted as the optimal rate, the % relative shortfall in using ACT = [74%‐65%)/74%] × 100% = 10.8%, indicating that approximately one in 10 of the patients in province who should have received ACT, did not actually receive it. Thus, the apparent shortfall between the provincial rate and the ABC benchmark is reduced by half when hospital‐specific rates of ACT are adjusted for patient characteristics.

## 4 DISCUSSION

In this study, we attempted to estimate the optimal rate of ACT utilization in stage III colon cancer. Several important findings have emerged. First, the ACT utilization rate in Ontario was 66%, but rates varied widely among hospitals. Monte Carlo modeling however showed that these rate variations were no greater than would be expected due to chance alone. Secondly, we found no evidence of systematic variations in ACT rates associated with health system‐related factors that might impede access to ACT. The adjusted ACT rate of 66% observed at comprehensive cancer centers was therefore accepted as the CBB benchmark rate. This was not significantly different from the overall ACT rate in Ontario, suggesting that there was no measurable shortfall in using ACT in Ontario. Thirdly, using the ABC approach, we identified a benchmark population which had an ACT rate of 81%; this would indicate that if this was adopted as the benchmark rate of ACT, during the study period, the shortfall was almost 20%. Adjusting the observed ACT rates for patient‐related factors resulted in a lower benchmark of 74%. The relative shortfall in ACT use is therefore approximately 10%, implying that one in 10 patients who needed ACT did not receive it. Collectively, these results illustrate the complexities in determining a benchmark rate for chemotherapy utilization and suggest that the ABC methodology may overestimate the need for ACT in this specific setting.

To interpret our findings, it may be useful to consider the assumptions that underlie any attempt to infer the appropriate rate of treatment from the distribution of observed rates. Common to the ABC and CBB methods is the assumption that observed subpopulation variations in treatment rates are, at least in part, a function of variation in practice patterns and access to care. However, the proportion of eligible patients seen at different institutions within the study period is subject to random variation. In attempting to infer the optimal rate from the distribution of institutional rates observed in a population, it is therefore critical to remember the contribution of random error, particularly when institutional sample sizes are small. This concern is equally important to the CBB and ABC methodologies.

The logic of the two benchmarking methods diverges at this point. The CBB method assumes that best practice is most likely to prevail at institutions that meet predetermined criteria for optimal access to treatment and optimal clinical decision‐making, and that the rates observed in those setting are likely to approximate the appropriate rate. The ABC method, as it was used in this study, assumes that high treatment rates are indicative of best practice and that, after taking some steps to eliminate institutions with very small numbers of cases, the rates observed at institutions that treat the highest proportion of cases are likely to approximate the appropriate rates.

Criterion‐based benchmarking has been widely used to estimate the need for radiotherapy and is used by policy makers to measure need and access to radiotherapy.^15^ Kong et al recently used this approach to identify a benchmark rate for palliative RT for brain metastases.^14^ Higher RT utilization was associated with more affluent neighborhood of residence, having on‐site RT facilities and closer proximity to RT facility. These factors were used to identify benchmark and non‐benchmark rates of 8% and 6% respectively, yielding a shortfall of 25%. In our study of colon cancer, we were unable to identify any system‐level barriers to use ACT and therefore assumed that the benchmark rate would approximate the rate seen in comprehensive cancer centers where there is facilitated access to multidisciplinary care. The most important finding of our study is that interhospital variation in ACT utilization rate is not significantly different from variation due to chance alone.

These findings have important implications for any effort to estimate benchmark rates for chemotherapy and for benchmarking methodology in general. The concept that smaller volume centers are more likely to be in the top 10% of performers simply due random variation is not often considered in benchmarking research. To our knowledge, no benchmarking methodology has evaluated the extent to which differences in performance rates between hospitals can be explained by random variation. Nonrandom variation suggests that there are systematic differences between providers and that those differences are modifiable; in theory this means that all providers should be able to achieve a benchmark performance rate. However, if differences in observed rates are due to random variation, as is suggested by the results of our current study, the calculated benchmark rate is more likely to be an artificial estimate of best performance. If our observation is confirmed in other independent datasets, policy makers should exercise great caution when considering whether or not to set a target performance rate using this methodology. We have recently performed a study to benchmark the rate of perioperative chemotherapy for muscle‐invasive bladder cancer (see companion paper). In this study, health system factors associated with chemotherapy were identified and we were therefore able to estimate an optimal rate of chemotherapy utilization using the criterion‐based benchmarking approach. In our ABC approach for bladder cancer, the Monte Carlo simulation demonstrated that the majority of observed variation was due to systematic (ie, nonrandom) variation. This has important implications for benchmarking in general and suggests that context is important in determining whether the ABC methodology is appropriate.

Our findings may be explained by the fact that ACT for stage III colon cancer has been widely accepted for many years and is relatively accessible across Ontario. It is therefore plausible that any differences may be based on chance alone. Future attempts to benchmark performance for quality indicators in other disease settings using these two methods may reveal significant systematic differences, especially if the treatment/intervention in question is less broadly accepted by the medical community and less accessible across all jurisdictions; this may explain the apparently divergent findings of our study in bladder cancer.

Our study should be interpreted in light of methodological limitations. Detailed information related to patient preferences, comorbidity, and performance status is not available. Our analysis is also limited to the study period (2002‐2008) and therefore may not reflect current ACT rates. It is possible that differences in case mix between hospitals/regions may explain some component of rate variation. To explore this we performed a multilevel multivariable logistic regression model and a parametric bootstrapping approach to derive “adjusted” benchmark rates; results of the intra‐class correlation coefficient suggested that ACT rate variation within hospitals was greater than variation between hospitals (Supplemental Appendix).

In summary, we have attempted to estimate a benchmark rate for ACT in stage III colon cancer using the criterion‐based and ABC methodology. As we did not observe any variation in ACT rates with geographic or socioeconomic factors, the benchmark rate could be assumed to be the ACT rate observed at comprehensive cancer centers. Our analysis highlights the potential pitfalls of using the ABC method for benchmarking. Without considering random vs systematic variation and by not adjusting for case mix, ABC‐derived benchmark rates are more likely to be an artificial estimate of best performance. Investigators and policy makers should be cautious in using ABC‐derived benchmark rates to set quality standards in other disease settings.

## CONFLICT OF INTEREST

The authors report no conflict of interest.

## Data Availability

The data that support the findings of this study are available from the Institute for Clinical Evaluative Sciences. Restrictions apply to the availability of these data, which were used under license for this study.
