# Supplementary figures and images for: Estimating the optimal rate of adjuvant chemotherapy utilization for stage III colon cancer
Source: Cancer Med. 2019 Aug 12;8(12):5590–9. doi: 10.1002/cam4.2456 (PMC6745837; doi:10.1002/cam4.2456)

**Supplemental eFigure 1. Identification of study cohort**

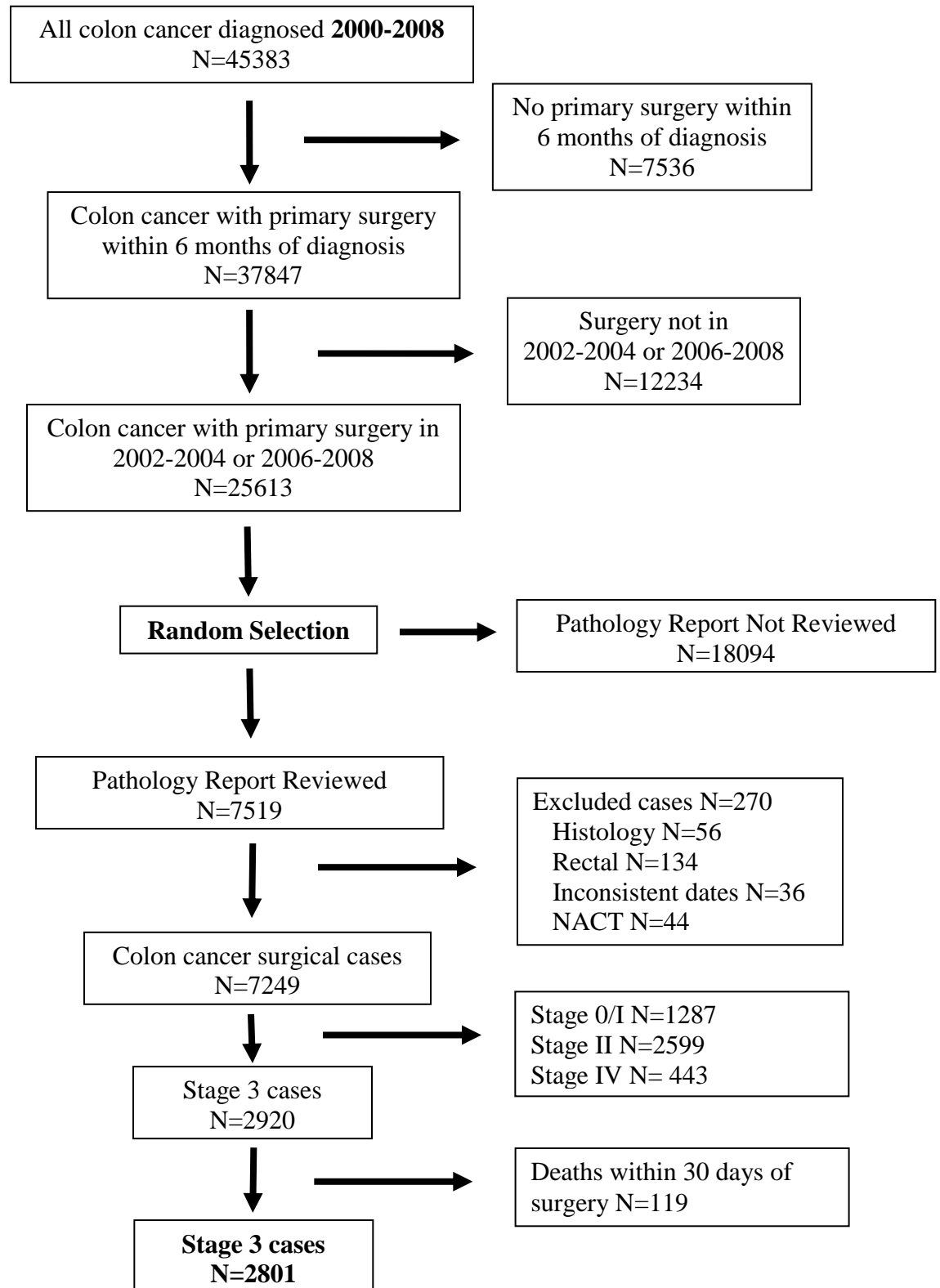

Supplement: Supplementary file 1 [file CAM4-8-5590-s001.pdf]
