# Supplementary material for: Estimating the optimal rate of adjuvant chemotherapy utilization for stage III colon cancer
Source: Cancer Med. 2019 Aug 12;8(12):5590–9. doi: 10.1002/cam4.2456 (PMC6745837; doi:10.1002/cam4.2456)
Supplement: Supplementary file 2 [file CAM4-8-5590-s002.pdf]

Supplemental eFigure 2. Adjusted hospital utilization rates of adjuvant chemotherapy for 2651 patients with stage III colon cancer treated in Ontario during 2002-2008.

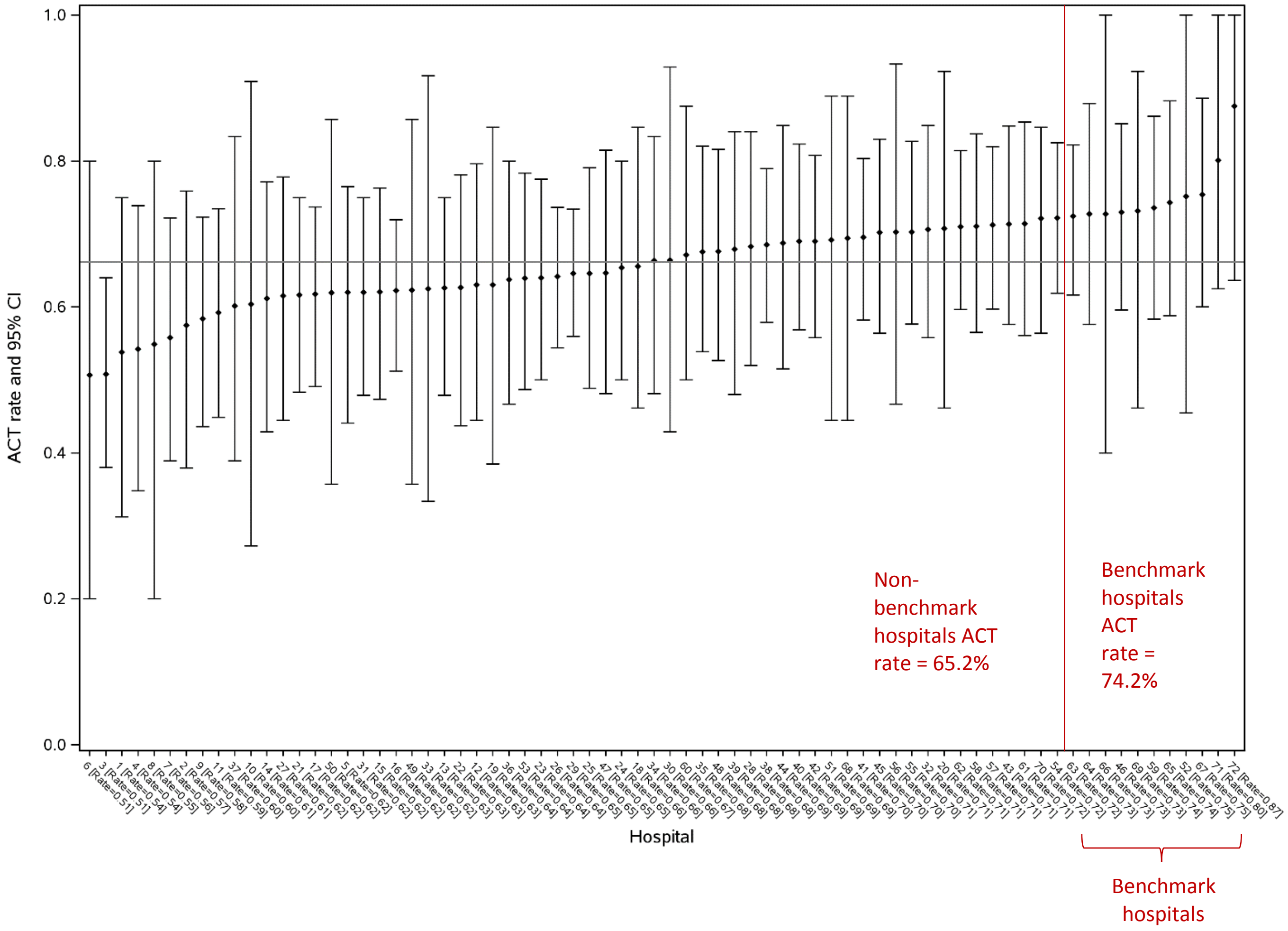

*Covariates adjusted for at the level of the patient include: age, SES, sex, charlson comorbidity, length of stay, T stage, N stage, LVI and grade. Rates of adjuvant chemotherapy and 95% CIs were obtained using a parametric bootstrapping approach consisting of 1000 simulations of the predicted probabilities for each patient from the multi-level multivariable regression model.

**Hospitals with patient volumes <10 were excluded from this figure (N=150 patients in total).
