# Supplementary material for: Estimating the optimal rate of adjuvant chemotherapy utilization for stage III colon cancer
Source: Cancer Med. 2019 Aug 12;8(12):5590–9. doi: 10.1002/cam4.2456 (PMC6745837; doi:10.1002/cam4.2456)
Supplement: Supplementary file 3 [file CAM4-8-5590-s003.docx]

**Supplemental eTable 1.** Characteristics of included vs excluded patients by surgical hospital patient volume

|  | All Patients | Patients operated on at hospitals with <10 patient volume | Patients operated on at hospitals with 10+ patient volume |
| --- | --- | --- | --- |
| Characteristic | N=2,801 | N=150 | N=2,651 |
|  | No. (%) | No. (%) | No. (%) |
| ***Patient-related*** |  |  |  |
| Age, years |  |  |  |
| 20-49 | 207 (7%) | 12 (8%) | 195 (7%) |
| 50-59 | 437 (16%) | 33 (22%) | 404 (15%) |
| 60-69 | 727 (26%) | 37 (25%) | 690 (26%) |
| 70-79 | 872 (31%) | 38 (25%) | 834 (31%) |
| 80+ | 558 (20%) | 30 (20%) | 528 (20%) |
| Sex |  |  |  |
| Female | 1,339 (48%) | 75 (50%) | 1,264 (48%) |
| Male | 1,462 (52%) | 75 (50%) | 1,387 (52%) |
| SES by quintile* |  |  |  |
| 1 | 595 (21%) | 34 (23%) | 561 (21%) |
| 2 | 648 (23%) | 48 (32%) | 600 (23%) |
| 3 | 590 (21%) | 33 (22%) | 557 (21%) |
| 4 | 509 (18%) | 24 (16%) | 485 (18%) |
| 5 | 453 (16%) | 11 (7%) | 442 (17%) |
| Unknown | 6 (0%) | 0 (0%) | 6 (0%) |
| Charlson comorbidity score |  |  |  |
| 0 | 2,267 (81%) | 117 (78%) | 2,150 (81%) |
| 1 | 313 (11%) | 22 (15%) | 291 (11%) |
| 2+ | 221 (8%) | 11 (7%) | 210 (8%) |
| ***Disease-related*** |  |  |  |
| Grade |  |  |  |
| Well-mod differentiated | 2,141 (76%) | 113 (75%) | 2,028 (76%) |
| Poor differentiated | 610 (22%) | 31 (21%) | 579 (22%) |
| Unstated | 50 (2%) | 6 (4%) | 44 (2%) |
| Lymphovascular invasion |  |  |  |
| Yes | 1,378 (49%) | 63 (42%) | 1,315 (50%) |
| No | 1,209 (43%) | 78 (52%) | 1,131 (43%) |
| NA | 214 (8%) | 9 (6%) | 205 (8%) |

| T stage |  |  |  |
| --- | --- | --- | --- |
| pT≤1 | 40-45 (2%) | ≤5 (2%) | 38-43 (2%) |
| pT2 | 178-183 (6%) | 10-15 (7%) | 165-170 (6%) |
| pT3 | 1,854 (66%) | 90 (60%) | 1,764 (67%) |
| pT4 | 724 (26%) | 47 (31%) | 677 (26%) |
| N stage |  |  |  |
| N1 | 1,663 (59%) | 95 (63%) | 1,568 (59%) |
| N2 | 1,138 (41%) | 55 (37%) | 1,083 (41%) |
| Lymph nodes harvest |  |  |  |
| Mean/Median | 17/15 | 15/13 | 17/15 |
| ≥12 | 2,051 (73%) | 91 (61%) | 1,960 (74%) |
| <12 | 740-745 (27%) | 59 (39%) | 685-690 (26%) |
| Unknown | <6 (0%) | 0 (0%) | <6 (0%) |

*Socioeconomic status, Quintile 1 represents the communities where the poorest 20% of the Ontario population resided. As per Institute of Clinical Evaluative Sciences policy, cells were suppressed to ensure that precise small cell values cannot be determined.

**Supplemental Appendix**

**COLON BENCHMARKING BOOTSTRAPPING APPROACH**

**METHODS**

To adjust ACT rates for case mix, a multi-level multivariable logistic regression model accounting for known patient- and disease-related characteristics associated with ACT and random variation at the level of hospital was employed. The predicted probability of each patient receiving ACT from the adjusted model is computed, and a new benchmark population is formed as patients from hospitals with top 10% average of the predicted probabilities. To estimate the ACT rates and its confidence intervals for the new benchmark and non-benchmark populations, we used a parametric bootstrapping approach with 1000 bootstrap samples to simulate ACT use using the predicted probability of each patient in the populations. The ACT rate and its 95% CI are obtained as the average and the 2.5th and 97.5th percentiles from 1000 bootstrap samples of ACT use in each population. Differences in hospital ACT rates were compared using logistic and modified poisson regression with the hospital as a fixed effect. We also calculated the intraclass correlation coefficient (ICC) to determine the degree of variation in ACT rates between hospitals compared to the variation within hospitals^[[1]](#footnote-1)^.

**RESULTS**

Subsequently, we adjusted the ACT rates by hospital for patient-and disease-related factors using the predicted probability for each patient and a parametric bootstrapping approach to identify an “adjusted hospital benchmark population”; by controlling for differences in case mix across hospitals this would be expected to reduce some of the apparently random variation in the use of ACT. The new benchmark population consisted of 319 patients from 11 hospitals (Supplemental eTable 2). The simulated ACT rate in this benchmark population was 74% (95% CI 70%-79%%) versus 65% (95% CI 63%-67%) in the non-benchmark population; these rates were significantly different using logistic and modified Poisson regression models (p<0.001 and p<0.001, respectively).The ICC in the unadjusted and adjusted models was 0.015 and 0.043 respectively, indicating that the ACT rate variation within hospitals is greater than the variation between hospitals.

**Supplemental eTable 2.** Characteristics of patients with stage III colon cancer treated in Ontario during 2002-2008 classified by hospital benchmark status after case mix adjustment.

| Characteristic | All Patients | Benchmark population | Non-Benchmark Population | P |
| --- | --- | --- | --- | --- |
|  | N=2,651 | N=319 | N=2,332 |  |
|  | No. (%) | No. (%) | No. (%) |  |
| ***Patient-related*** |  |  |  |  |
| Age (years) |  |  |  | 0.259 |
| 20-49 | 195 (7%) | 28 (9%) | 167 (7%) |  |
| 50-59 | 404 (15%) | 47 (15%) | 357 (15%) |  |
| 60-69 | 690 (26%) | 91 (29%) | 599 (26%) |  |
| 70-79 | 834 (31%) | 103 (32%) | 731 (31%) |  |
| 80+ | 528 (20%) | 50 (16%) | 478 (20%) |  |
| Sex |  |  |  | 0.558 |
| Female | 1,264 (48%) | 157 (49%) | 1,107 (47%) |  |
| Male | 1,387 (52%) | 162 (51%) | 1,225 (53%) |  |
| SES by quintile* |  |  |  | 0.002 |
| 1 | 561 (21%) | 45 (14%) | 516 (22%) |  |
| 2 | 600 (23%) | 76 (24%) | 524 (22%) |  |
| 3 | 557 (21%) | 60 (19%) | 497 (21%) |  |
| 4 | 485 (18%) | 79 (25%) | 406 (17%) |  |
| 5 | 442 (17%) | 59 (18%) | 383 (16%) |  |
| Unknown | 6 (0%) | 0 (0%) | 6 (0%) |  |
| Charlson comorbidity score |  |  |  | 0.361 |
| 0 | 2,150 (81%) | 266 (83%) | 1,884 (81%) |  |
| 1 | 291 (11%) | 34 (11%) | 257 (11%) |  |
| 2+ | 210 (8%) | 19 (6%) | 191 (8%) |  |
| Length of hospital stay (days) |  |  |  |  |
| Median | 8 | 8 | 8 | <0.001 |
| ***Disease-related*** |  |  |  |  |
| Grade |  |  |  | 0.445 |
| Well-mod differentiated | 2,028 (76%) | 253 (79%) | 1,775 (76%) |  |
| Poor differentiated | 579 (22%) | 60-65 (19%) | 515-520 (22%) |  |
| Unstated | 44 (2%) | ≤5 (2%) | 35-40 (2%) |  |
| Lymphovascular invasion |  |  |  | 0.466 |
| Yes | 1,315 (50%) | 158 (50%) | 1,157 (50%) |  |
| No | 1,131 (43%) | 131 (41%) | 1,000 (43%) |  |
| NA | 205 (8%) | 30 (9%) | 175 (8%) |  |

| T stage |  |  |  | 0.010 |
| --- | --- | --- | --- | --- |
| pT≤1 | 40 (2%) | 8 (3%) | 32 (1%) |  |
| pT2 | 170 (6%) | 30 (9%) | 140 (6%) |  |
| pT3 | 1,764 (67%) | 216 (68%) | 1,548 (66%) |  |
| pT4 | 677 (26%) | 65 (20%) | 612 (26%) |  |
| N stage |  |  |  | 0.600 |
| N1 | 1,568 (59%) | 193 (61%) | 1,375 (59%) |  |
| N2 | 1,083 (41%) | 126 (39%) | 957 (41%) |  |
| Lymph node harvest |  |  |  | 0.084 |
| ≥12 | 1,960 (74%) | 243 (76%) | 1,717 (74%) |  |
| <12 | 685-690 (26%) | 70-75 (23%) | 611-616 (26%) |  |
| Unknown | ≤5 (0%) | ≤5 (1%) | ≤5 (0%) |  |

*Socioeconomic status, Quintile 1 represents the communities where the poorest 20% of the Ontario population resided.

As per Institute of Clinical Evaluative Sciences policy, cells were suppressed to ensure that precise small cell values cannot be determined.

1. - Snijders T.A.B, Bosker RJ. Multilevel Analysis: An introduction to basic and advacned multilevel modelling. London: Sage Publishers 2011. [↑](#footnote-ref-1)
